# Differential Effects of Workaholism and Work Engagement on the Interference Between Life and Work Domains

**DOI:** 10.5964/ejop.v14i4.1626

**Published:** 2018-11-30

**Authors:** Giovanni Di Stefano, Maria Gaudiino

**Affiliations:** a Department of Psychology, Educational Science & Human Movement, University of Palermo, Palermo, Italy; Department of Psychology, Webster University Geneva, Geneva, Switzerland; University of Wollongong, Wollongong, Australia

**Keywords:** workaholism, work engagement, work–life interference, work-to-life conflict, life-to-work conflict

## Abstract

This study analyzed the differences between workaholism and work engagement in relation to their influence on work–life interference. Workaholism is an addiction to work, characterized by obsessive attitude towards job, whereas work engagement concerns a positive pattern of thoughts and feelings about one’s job; these two constructs thus represent pathological and healthy forms of heavy work investment, respectively. As a consequence, it was expected that workaholism and work engagement would have different effects on perceived interference between work and life domains. We assessed levels of workaholism, work engagement, work-to-life interference, and life-to-work interference in a sample of 212 Italian workers. Results from structural equation modeling showed an inverse symmetry involving patterns and magnitudes of the relations observed: work engagement was more negatively related to life-to-work interference than work-to-life interference, whereas workaholism was more positively related to work-to-life interference than life-to-work interference. Implications about findings of the study are discussed.

Workaholism and work engagement are studied in literature as two different ways of involvement in work activity, with distinctive outcomes affecting in turn life roles and well-being, in both work and nonwork domains of life (Shimazu & Schaufeli, 2009).

The concept of workaholism appears several decades ago, introduced by Oates (1971), and it is subsequently developed from different perspectives. Porter (1996) defines workaholism “…as excessive involvement with work evidenced by neglect in other areas of life and based on internal motives of behavior maintenance rather than requirements of the job or organization” (p. 71), thus underlining the inner compelling drive that pushes workaholics to strenuously work (Spence & Robbins, 1992). Although from an external point of view workaholic behavior might just be seen as hard working or overwork (Del Líbano Miralles, 2011; Douglas & Morris, 2006; Scott, Moore, & Miceli, 1997), the excessive amount of time and energy dedicated to work is not justified by need for money or organizational demands (Harpaz & Snir, 2003), but motivated by something workaholics feel inside, a compulsion towards work (Robinson, 1998); consequently, as stated by Machlowitz (1980), a simple count of working hours is not an adequate strategy to differentiate workaholics from other workers. Among the other conceptualizations (e.g., Naughton, 1987; Scott et al., 1997), Spence and Robbins’ (1992) “workaholic triad” model has received major attention in literature. According to Spence and Robbins (1992), workaholism is characterized by the dimensions of work involvement, drive, and low enjoyment which lead workaholics to feel they cannot stop their job activities, experiencing a deep sense of pressure, distress, and guilt when not working. More recently, work addiction has been described as resulting from the dimensions of working excessively and working compulsively (Schaufeli, Taris, & Bakker, 2006, 2008), with the former indicating workaholics’ long working hours and the latter underlining the internal feeling of obligation towards work.

Following Schaufeli, Salanova, González-Romá, and Bakker (2002), the second type of work attitude, namely work engagement, is a “positive, fulfilling, work-related state of mind” (p. 74) that leads individuals to intensively work long hours, being devoted to their job at the same time.

Since the outcomes expected from workaholism and work engagement on people’s life domains significantly differ (Clark, Michel, Stevens, Howell, & Scruggs, 2014; Shimazu & Schaufeli, 2009; Shimazu, Schaufeli, Kubota, & Kawakami, 2012), especially when considering the adjustment of energy and time an individual addresses to each life spheres (Kirchmeyer, 2000), the concept of work–life interface may have a critical role in making a difference between workaholics’ and engaged workers’ experience. Surprisingly, this difference is not clearly highlighted in literature or in the field (Douglas & Morris, 2006; Friedman & Lobel, 2003), with the practical risk of a dangerous confusion concerning models of work behaviors: for example, promoting heavy investment in a job could seem a profitable occasion to increase production in organizations, but this naive idea shows a lack of awareness of all the negative outcomes implied by an exaggerate involvement in work. Coherently, we believe that, if different types of such deep work investment entail so different repercussions in human life, it is crucial to try to understand their relation and refine the knowledge about them.

The present study therefore aims to investigate the construct of interference between work and life domains (the negative side of work–life interface) (Frone, 2003) as a possible outcome that allows to differentiate healthy and problematic heavy work investment (Snir & Harpaz, 2012), useful in both theoretical and practical point of view.

## Healthy and Pathological Involvement in Work: The Role of Work–Life Interface

The comparison between workaholism and work engagement can be viewed from two broad perspectives: the first considers the *underlying dimensions* of the two constructs (e.g., Bakker & Oerlemans, 2011; Schaufeli et al., 2002); the second takes into account the *outcomes* resulting from them (e.g., Clark et al., 2014; Matuska, 2010).

From the first perspective, it is possible to disentangle the differences between workaholism and work engagement by paying attention to their distinctive nature and composition, and measuring the intensity of the attitudes they imply. As anticipated above, the most cited models used in research to study workaholism and work engagement are both proposed by Schaufeli and colleagues (e.g., Schaufeli et al., 2002; Schaufeli, Taris, & Bakker, 2006). In particular, on the one hand, workaholism is analyzed through a two-dimensional structure, consisting of *working excessively* and *working compulsively* (Schaufeli, Taris, & Bakker, 2008), according to which the former indicates an effort made at job that exceeds the normal request, whereas the latter involves the inner sense of obligation perceived towards working, even in obsessive terms. On the other hand, work engagement is measured through a three-dimensional model, including: *vigor*, the intense energy used to sustain hardworking; *dedication*, the sense of pride and importance related to doing a certain job; and *absorption*, the condition under which workers are fully immersed in their activity (Schaufeli, Bakker, & Salanova, 2006; Schaufeli et al., 2002). Interestingly, Schaufeli, Taris, and Bakker (2006) found a correlation between work engagement and the subdimension of working excessively, but not between work engagement and working compulsively, so that compulsion could be seen as the discriminating element of workaholic behavior. Another point of distinction concerns the nature of emotions elicited by workaholism and work engagement (Gorgievski & Bakker, 2010; van Wijhe, Peeters, & Schaufeli, 2011), particularly in relation to fun, which is considered a typical experience of engaged workers, not of workaholics (Bakker, Shimazu, Demerouti, Shimada, & Kawakami, 2013; Taris, Schaufeli, & Shimazu, 2010). According to the circumplex model (Russell, 1980), workaholism could be described as a phenomenon characterized by high activation and low pleasure, and work engagement as characterized by high level of both activation and pleasure (Bakker & Oerlemans, 2011; Dijkhuizen, Veldhoven, & Schalk, 2016). Furthermore, Griffiths and Karanika-Murray (2012) propose a continuum of engagement, where workaholism is placed on the highest pole (extreme engagement), whilst work engagement is located in the middle area (healthy engagement), between extreme engagement and the lowest pole of the continuum (withdrawal).

With regard to similarities, the dimension of absorption identifies to a certain extent an overlapping area between workaholism and work engagement, since it seems to catch the difficulty to stop working and dedicate oneself to nonwork activities (Del Líbano, Llorens, Salanova, & Schaufeli, 2012; Schaufeli et al., 2002; Schaufeli, Taris, & van Rhenen, 2008). All these contributions help to understand the conceptual basis and the individual experience of being a workaholic or an engaged worker.

A second, different point of view used in comparing workaholism and work engagement underlines the *outcomes* of individuals’ work attitude on well-being and social life domains. Work engagement is characterized by low levels in health complaints (Hallberg & Schaufeli, 2006) and high levels in in-role and extra-role performance and well-being (Bailey, Madden, Alfes, & Fletcher, 2017); further, it is related to job and personal resources, so that engaged employees are more likely to perceive themselves as self-efficacious, optimistic, and important for their organization, in comparison to nonengaged colleagues (Xanthopoulou, Bakker, Demerouti, & Schaufeli, 2009). Conversely, research shows that workaholism is associated with low job and career satisfaction (Burke & MacDermid, 1999) and low levels of life satisfaction, whereas it is positively related to physical health problems and strain (Clark, Michel, Zhdanova, Pui, & Baltes, 2016). Moreover, as reported by Ng, Sorensen, and Feldman (2007), workaholism is characterized by poor mental health and social relationships, and poor job performance in the long run.

Among the outcomes, in the last decades, research has increasingly focused on the concept of work–life interface (Greenhaus, Collins, & Shaw, 2003; Matuska, 2010), the configuration which takes shape from the relations between work role and other roles in life (Marks & MacDermid, 1996). We cannot find a unique definition of work–life interface: for example, Demerouti and colleagues (Demerouti, Bakker, & Voydanoff, 2010; Demerouti, Geurts, & Kompier, 2004) define it as a process in which the functioning and behavior in one role is influenced by demands and resources from the other role. Nevertheless, this concept covers multiple facets concerning what people perceive and experience when taking into account the relation established between their work and nonwork life: indeed, it is possible to distinguish the topics of balance, enrichment, and conflict between family and work domains. Kirchmeyer (2000) suggests that work–life balance is linked to a satisfying distribution of personal resources (e.g., energy, time) across all life domains, so that we expect that individuals experience positive thoughts and feelings in relation to those domains. In contrast, enrichment is a process by which the participation in one domain strengthens or improves the quality of the participation in the other one (Ghislieri, Martini, Gatti, & Colombo, 2011). Finally, in the case of work–life conflict, a lack of such equilibrium in people’s lives might derive from an incompatibility between two or more domains and provoke potential harmful outcomes on individual well-being, owing to the pressure perceived from conflicting role requirements (Burke & El-Kot, 2010).

The greatest part of researchers’ interest was directed towards conflict resulting from a negative interaction among life domains (Grawitch, Barber, & Justice, 2010; Greenhaus & Beutell, 1985; Kossek & Ozeki, 1998), while more recently several studies have addressed the positive side of balance, concerning the effect of facilitating or enhancing multiple investment in roles (Ghislieri et al., 2011; Grawitch et al., 2010; Grzywacz & Butler, 2005). Scholars have investigated positive and negative aspects of work–life interface as results of balance and conflict, respectively, between work and nonwork spheres; they focused in particular on balance between work and family domains (Demerouti, Taris, & Bakker, 2007; Frone, 2003) and introduced a finer specification of the direction in which the involvement in one of the two domains can affect the participation in the other (Amstad, Meier, Fasel, Elfering, & Semmer, 2011; Netemeyer, Boles, & McMurrian, 1996). From this point of view, we can refer to conflict “from work to family” in order to indicate when interference occurring in one’s family role is caused by work activities and, vice versa, we can refer to conflict “from family to work” in order to specify that demands from family hinder work task completion.

Since workaholism and work engagement both concerns an intense working style, work–life interference stands out among other possible outcomes and appears as particular relevant to investigate whether the two work attitudes can be distinguished in the field.

Workaholics’ behavior is characterized by tendency to indulge in work activities, exceeding specific organizational demands (Porter, 1996), due to the compulsive constraint they feel (Del Líbano Miralles, 2011; Schaufeli & Salanova, 2011; Spence & Robbins, 1992), so we expect that such behavior can lead to a consistent depletion of individual resources over time (Bakker, Demerouti, Oerlemans, & Sonnentag, 2013; van Wijhe, Peeters, Schaufeli, & Ouweneel, 2013); as a consequence, multiple life spheres are likely to be neglected and perceived like a source of interference for work-related tasks, increasing the risk of work–life interference and reducing the possibility of a positive interplay (Bakker, Demerouti, & Burke, 2009; Bonebright, Clay, & Ankenmann, 2000; Shimazu & Schaufeli, 2009; Taris, Schaufeli, & Verhoeven, 2005). Consequently, our first hypothesis is:

Hypothesis 1. *Workaholism is positively related to work-to-life and life-to-work interference.*

On the other hand, work engaged people express their passion for work in a healthier way (Griffiths & Karanika-Murray, 2012), working long hours as workaholics do, but without the sense of compulsory obligation and with a strong feeling of enjoyment and fun (Del Líbano Miralles, 2011; Schaufeli & Salanova, 2011; Taris, Schaufeli, & Shimazu, 2010). Engaged workers report high job and life satisfaction (Christian, Garza, & Slaughter, 2011; Schaufeli, Taris, & Bakker, 2006; Schaufeli, Taris, & van Rhenen, 2008; van Beek, Hu, Schaufeli, Taris, & Schreurs, 2012) and more positive emotions than workaholics (Clark et al., 2014; van Wijhe et al., 2011), so they seem to possess personal and job resources that can protect them (Burke & El-Kot, 2010; Greenhaus & Powell, 2006; Xanthopoulou et al., 2009), when confronting resources draining processes triggered by involvement in multiple life roles (Amstad et al., 2011; Grawitch et al., 2010). Although some authors posit engagement as a positive outcome of interactions between work and nonwork domains (e.g., Li, Zhong, Chen, Xie, & Mao, 2014; Montgomery, Peeters, Schaufeli, & Ouden, 2003; Timms et al., 2015), there are several models that emphasize the impact work engagement directly has on work–life interface (Amstad et al., 2011; Bakker & Demerouti, 2013). We therefore hypothesize:

Hypothesis 2. *Work engagement is negatively related to work-to-life and to life-to-work interference.*

However, as reported before, workaholism and work engagement show at least a common area in relation to the excessive amount of time spent in job activity (Schaufeli, Taris, & Bakker, 2006) and the difficulty to detach from it (Schaufeli, Taris, & van Rhenen, 2008). This partial overlapping leads to expect that:

Hypothesis 3. *Workaholism and work engagement are weakly and positively related.*

Finally, despite the fact that the two directions of interference between work and nonwork domains are conceived and examined through distinctive constructs (Netemeyer et al., 1996), findings from research show they are positively related (e.g., Greenhaus et al., 2003; Kinnunen, Feldt, Mauno, & Rantanen, 2010). It seems that people reporting experience of work–life interference perceive to a certain extent both work-to-life and life-to-work conflict, although they distinguish their levels. So, a positive relation between the two directions of work–life interference is also predicted:

Hypothesis 4. *Work-to-life interference and life-to-work interference are positively related.*

**Figure 1:**
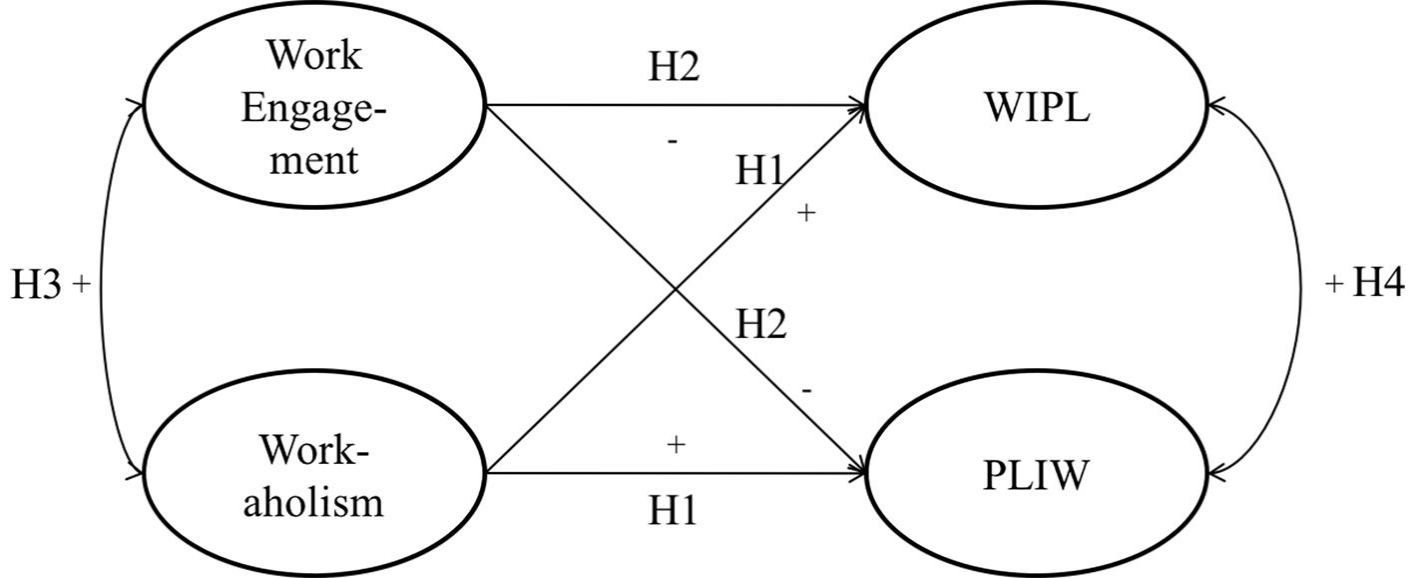
Theoretical model of the study. *Note.* WIPL = Work Interference to Personal Life; PLIW = Personal Life Interference to Work.

The hypothetical model defined in this study is depicted in Figure 1.

## Materials and Method

### Participants and Procedure

This study was carried out with 212 Italian adults working in different organizations from different professional sectors (banking, health, food, industry, commerce). They were aged 18 to 66 years (*M* = 42.61, *SD* = 11.01) and 53.1% were men; 63.6% had a permanent contract and the organizational tenure was 1 to 40 (*M* = 17.99, *SD* = 10.39). To qualify for inclusion in our study, participants needed to work full time. We sent letters explaining the study, guaranteeing confidentiality and defining the requirements for inclusion to 300 employees (response rate was 70.7%) working in different organizations. Questionnaires in paper format were individually completed at the workplace. To reduce common method bias (Podsakoff, MacKenzie, Lee, & Podsakoff, 2003), the survey was anonymous and had its item orders counterbalanced across the organizations participating in the study.

### Measures

*Work engagement* was measured by the Italian validation (Balducci, Fraccaroli, & Schaufeli, 2010) of UWES-9 (Schaufeli, Bakker, & Salanova, 2006), the 9-item version of Utrecht Work Engagement Scale (Schaufeli & Bakker, 2003). It consists of three scales reflecting work engagement components (i.e., Vigor [VI], Dedication [DE], and Absorption [AB]), containing 3 items each. Example items are: “At my job, I feel strong and vigorous” (VI), “My job inspires me” (DE), “I get carried away when I am working” (AB). Participants answered how often they had experienced specific work-related feelings and states, according to a 7-point frequency scale from 1 (*Never*) to 7 (*Always*).

*Work–life interference* was measured by an Italian adaptation (Pipitone, 2015) of Hayman’s (2005) instrument. In accordance to the specific interest of this study, we included only the two scales measuring the conflicting facet of balance, thus excluding Work/Personal Life Enhancement scale. The instrument used contained 11 items: 7 items from Work Interference with Personal Life scale (WIPL; e.g., “My job makes my personal life difficult”), and 4 items for Personal Life Interference with Work scale (PLIW; e.g., “My personal life drains me of energy for work”). All the items were scored on a 7-point frequency Likert scale, from 1 (*Never*) to 7 (*Always*), in order to indicate how much participants had experienced the situations described by the statements, during the previous three months.

*Workaholism* was assessed by the Italian validation (Balducci, Avanzi, Consiglio, Fraccaroli, & Schaufeli, 2017) of Schaufeli, Taris, and Bakker’s (2006) Dutch Work Addiction Scale (DUWAS). The instrument consists of two scales, Working Excessively (WE) and Working Compulsively (WC), containing 9 and 6 items, respectively. The former derives from Flowers and Robinson’s (2002) Compulsive Tendency scale and refers to working hard, neglecting nonwork activities, staying busy over time; the latter derives from Spence and Robbins’ (1992) Drive scale, is included in their measure of workaholism (Workaholism Battery: WorkBat), and evaluates the sense of obligation and guilt that pushes workaholics to work and their difficulties to relax. Example items are “I find myself doing two or three things at one time such as eating lunch and writing a memo, while taking on the telephone” (WE) and “I feel that there’s something inside me that drives me to work hard” (WC). Subjects answered how often they had experienced the situations described by each item, by choosing an alternative on a Likert scale, from 1 (*Never*) to 6 (*Very often/Always*).

## Results

Table 1 shows the descriptive statistics of the variables and their zero-order correlations. As Table 1 shows, virtually all correlations were in the expected direction. At first glance, the patterns of correlation of work engagement and workaholism with work-to-life conflict were similar, albeit we observed different magnitudes and opposite directions (*r* = -.17, *p* < .05, and *r* = .36, *p* < .01, respectively). Furthermore, also the patterns of correlation of work engagement and workaholism with life-to-work conflict were similar, but with different signs (*r* = -.35, *p* < .01, and *r* = .16, *p* < .05, respectively). Gender, age, and organizational tenure were unrelated to study variables and were therefore not included as control variables in subsequent model testing.

**Table 1:** Means (M), Standard Deviations (SD), Internal Consistencies (Cronbach’s α), and Zero-Order Correlations of the Study Variables (N = 212)

Variables		*M*	*SD*	α	1	2	3	4	5	6	7	8	9	10	11
1.	*Gender*	0.47	0.50	-											
2.	*Age*	42.61	11.01	-	-.106										
3.	*Tenure*	17.99	10.39	-	-.269**	.839**									
4.	*WIPL*	3.72	1.60	.92	.040	.052	.081								
5.	*PLIW*	2.69	1.09	.73	-.020	.011	.063	.343**							
6.	*W-EN*	5.22	1.34	.94	.078	-.009	-.032	-.170*	-.348**						
7.	DE	5.13	1.53	.86	.110	-.069	-.072	-.177*	-.325**	.920**					
8.	AB	5.46	1.34	.90	.060	.073	.026	-.168*	-.278**	.895**	.706**				
9.	VI	5.06	1.50	.80	.044	-.020	-.038	-.280**	-.353**	.946**	.819**	.787**			
10.	*WO*	3.95	1.02	.82	.031	.053	.049	.363**	.160*	.285**	.189**	.328**	.279**		
11.	WE	3.77	1.03	.75	.043	.043	.090	.481**	.192*	.286**	.234**	.286**	.274**	.813**	
12.	WC	4.13	1.34	.80	.014	.047	.006	.182**	.020	.213**	.117*	.279**	.213**	.895**	.466**

*Note*. WIPL = Work Interference with Personal Life; PLIW = Personal Life Interference with Work; W-EN = Work Engagement; DE = Dedication; AB = Absorption; VI = Vigor; WO = Workaholism; WE = Working Excessively; WC = Working Compulsively. Gender was coded as a dummy variable (male = 0; female = 1).

*p < .01. **p < .001.

In order to test our hypotheses, structural equation modeling method with latent variables was used, as implemented by AMOS 20 (Arbuckle, 2011). To evaluate the goodness of fit of the theoretical model to the data, maximum likelihood estimation was used and the input for the analysis was the covariance matrix of the items. The goodness-of-fit of the model was evaluated using the χ^2^ goodness-of-fit statistic and the Root Mean Square Error of Approximation (RMSEA). Two relative goodness-of-fit indices were also computed: the Non-Normed Fit Index (NNFI) and the Comparative Fit Index (CFI). For both relative-fit indices, as a rule of thumb, values greater than .90 are considered as indicating a good fit (Byrne, 2010), whereas values smaller than .08 for RMSEA indicate an acceptable fit (Cudeck & Browne, 1993). The tested model showed a good fit to the data, with all the fit-indices meeting their respective criterion: χ^2^ = 226.45, *df* = 98, *p* < .0000, χ^2^/*df* ratio = 2.31, RMSEA = .07, NNFI = .91, CFI = .93.

**Figure 2:**
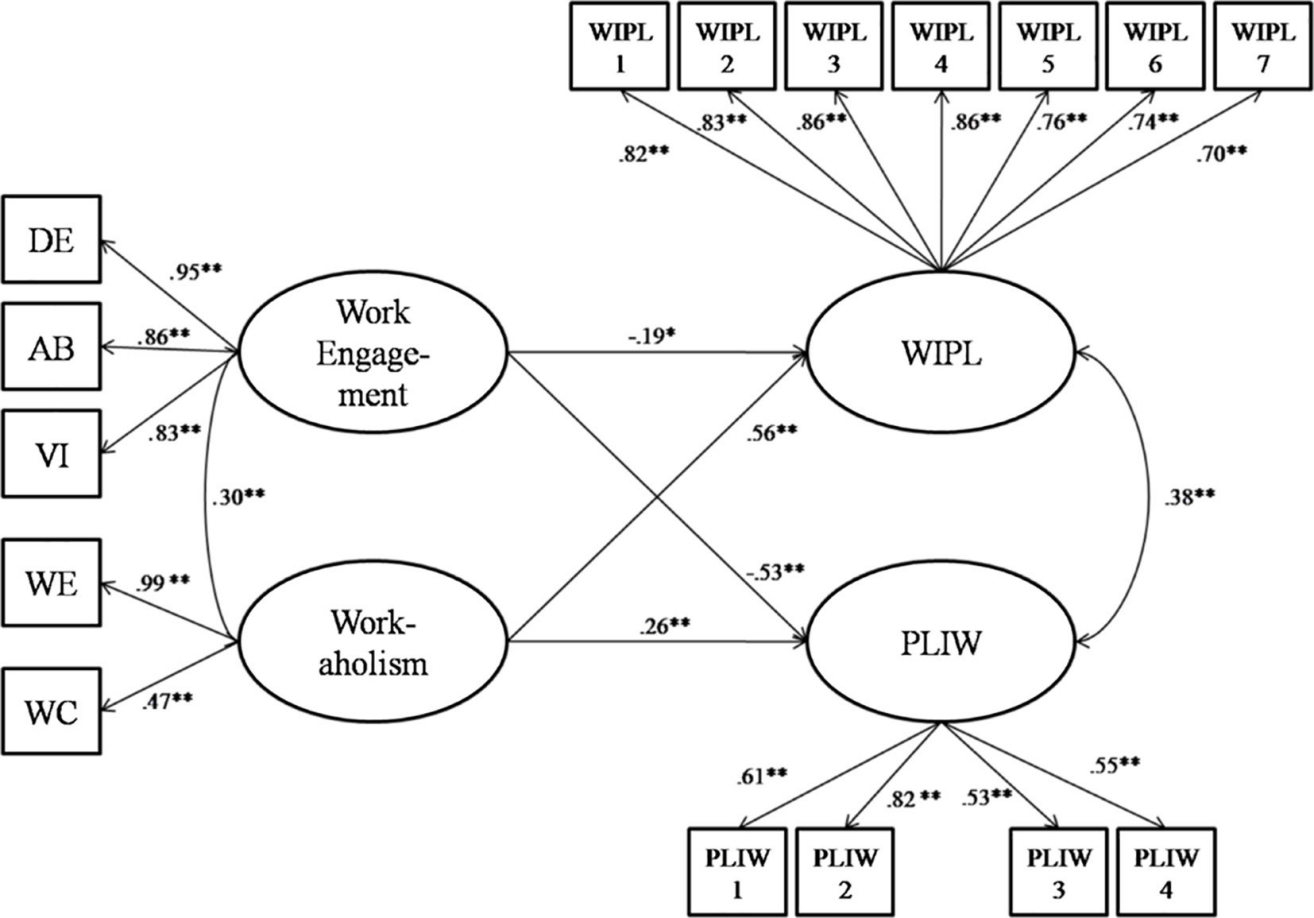
The relations between work engagement, workaholism, work-to-life conflict and life-to-work conflict. *Note.* WIPL = Work Interference to Personal Life; PLIW = Personal Life Interference to Work; DE = Dedication; AB = Absorption; VI = Vigor; WE = Working Excessively; WC = Working Compulsively. For parsimony, latent variable errors are omitted. *p < .01. **p < .001.

As can be seen from Figure 2, work engagement strongly and negatively loaded on life-to-work interference (Hypothesis 2: γ = -.53, *p* < .001), whereas workaholism loaded less and in the opposite direction (Hypothesis 1: γ = .26, *p* < .001); symmetrically, consistent with Hypothesis 1, workaholism strongly and positively loaded on WIPL (γ = .56, *p* < .001), while, consistent with Hypothesis 2, work engagement loaded less and negatively (γ = -.19, *p* < .01).

Finally, Figure 2 further shows, as expected, that the latent work engagement and workaholism factors were positively correlated (ϕ = .30, *p* < .001), thus confirming Hypothesis 3, whereas WIPL was positively correlated with PLIW (Hypothesis 4: ψ = .38, *p* < .001).

## Discussion

The objective of the current study was to examine two specific approaches to work in relation to the degree of interference between work and nonwork life of individuals. Among all the possible outcomes to consider in order to study these associations, we chose work–life interference because workaholism and work engagement both point to an intense working style (Bakker, Shimazu, et al., 2013; Schaufeli, Taris, & Bakker, 2006), so levels of conflict perceived by individuals between work and nonwork domains can represent a sign of imbalance in one’s life (Aziz & Cunningham, 2008), allowing to identify potential cases of pathological investment in work, namely workaholism.

Our first point of attention was the relation between workaholism and work–life interference, for which findings confirmed a positive correlation, when observed either from work domain to personal life domain or from personal life domain to work domain. Workaholics’ obsessive attitude towards work hampers their opportunity to equally allocate time and energy to all their life spheres, so work drains their available resources (van Wijhe et al., 2013) and they consequently report a high conflicting perception of work and nonwork roles (Burke & El-Kot, 2010; Taris, Schaufeli, & Verhoeven, 2005), in comparison to engaged workers.

On the contrary, as predicted, work engagement showed a negative relation with work–life interference, both for work-to-life and for life-to-work directions. Engaged workers spend a large amount of time at work, seemingly to what happens for workaholics (Schaufeli & Salanova, 2011; van Wijhe, Peeters, & Schaufeli, 2011), but with a substantial difference illustrated just by their level of balance perceived in personal life: in this case, deep investment in work does not prevent them from engaging in various roles other than work role (Clark et al., 2014; Timms et al., 2015). Hence, their passion for work is compatible with their different interests or routine activities.

Interestingly, workaholism was positively associated with interference, while work engagement showed a negative correlation with it, but, further, each type of investment in work had the strongest relation with a different direction of interference. Precisely, the link between workaholism and work-to-life interference was stronger than the link between workaholism and life-to-work interference, whereas the relation between work engagement and work-to-life interference was weaker than the relation between work engagement and life-to-work interference.

With regard to the pattern of results emerged for workaholism, an explanation could be offered by the sense of constraint workaholics feel towards the action of working. Although they might seem simply devoted to their job (Douglas & Morris, 2006), they experience work as an obsession, an obligation they are pushed to (Spence & Robbins, 1992), so it is perceived as something they cannot avoid. Workaholics spend all their efforts in work activities, so they could be more likely to attribute to work, rather than to another side of their lives, the cause of lacking care about private and social life, the cause of difficulties in conciliating tasks from various roles, and even the cause of neglecting personal needs and well-being. Since their time is dominated by need for work, it is plausible that workaholics feel work, more than nonwork domain, as the deleterious element of imbalance in life. Moreover, our results can be seen in line with the prevalence of conflict from work to life, in respect to the opposite direction of interference (Frone, 2003; O’Driscoll, Ilgen, & Hildreth, 1992).

Moving attention to work engagement, a similar pattern was found by Grawitch, Barber, and Justice (2010): engaged workers are inclined to perceive lower levels of work-to-life interference, probably since they are not obsessed with work, differently from work addicts (Schaufeli, Shimazu, & Taris, 2009), although engaged workers are deeply involved in their job. However, they feel passionate about their work, not tantalizingly obliged to it.

On the other hand, work engagement is much more strongly associated with lower levels of life-to-work interference, so it can be worthwhile to reflect upon the role played by nonwork context for engaged workers. These individuals do not appear to consider private life duties as obstacles to their work role and they seldom feel that private life depletes their energies, making them too tired for working. Rather, the lower scores on life-to-work interference reported in this study by engaged subjects could suggest that time spent in nonwork domains has great importance, for them, and contributes to building a satisfying balance among all domains. This may correspond, to a certain extent, to the asymmetry O’Driscoll et al. (1992) found between pressures exerted by demands from job domain and pressures exerted by demands from off-job domain in respect to the interferences aroused each to the complementary domain: it seems that a greater off-job time pressure does not predict a greater off-job interference; on the contrary, a greater job time pressure predicts a greater job interference. As a consequence, the hours dedicated to life spheres other than work could contribute to replenishing of self-regulation resources of those workers who are engaged in their job (Barber, Grawitch, & Munz, 2013).

Finally, in our study, as emerged in some previous research also (Shimazu & Schaufeli, 2009; Shimazu et al., 2012), workaholism and work engagement showed, to a certain extent, a positive relation, which supports the idea that, although they are two different constructs, there is an overlapping portion at the basis of their meaning. Moreover, as expected, and in line with previous research (Greenhaus et al., 2003; Kinnunen, Feldt, Mauno, & Rantanen, 2010), the present study showed a positive relation between the two different forms of interference (i.e., work-to-life and life-to-work).

## Conclusion, Limitations, Future Research and Practical Implications

In the current study we examined the relations between workaholism and work engagement, on the one hand, and between these two work attitudes, respectively, and work–life interference, on the other hand. Findings confirmed the expectation of a differential sign of such relations: workaholism was positively associated with work–life conflict, in both work-to-life and life-to-work directions, while work engagement was negatively associated with both directions of conflict between work and life. More in detail, we further observed a reversed symmetry among the relations of the two work attitudes respectively with each of the two directions of conflict, that is we found that workaholism showed a stronger positive correlation with work-to-life conflict, and work engagement showed a stronger negative correlation with life-to-work conflict. These results raised interesting questions that should be addressed by future research on the theme. Finally, workaholism and work engagement are different approaches to work, but they are likely to share a portion of their conceptual dimensions.

The study presented here shows some limitations. First, it suffers from the typical limits of research based on self-report measures. All data are based on self-reports, which means that the magnitude of the effects that we reported may have been biased due to common method variance (Conway, 2002; Podsakoff et al., 2003). Future research could include more objectively measured variables. Second, cross-sectional nature of the study prevents us from advancing any conclusions about causal relations among the variables we investigated. Third, the sample consists of a relatively homogeneous small group of people who come from a delimited geographical area; this might have led to a restriction of range. Hence, future research should include more heterogeneous samples, thus increasing the generalizability of the results. However, there are some points emerged from this study that potentially stimulate new research questions. In particular, we refer to the possibility to deepen the research on the distinctive associations of workaholism and work engagement, as two types of work investment, with bidirectional work–life balance, since, as just suggested by our study, each attitude may determine significant differences not only in the amount of perceived conflict, but also in its nature or direction, that is its source. We conclude that common and distinctive components of workaholism and work engagement deserve further attention, by focusing, for example, on other variables that could be conceptualized as correlated with these peculiar approaches to work.

Moreover, this research may suggest some implications for managerial practice, by highlighting how two different types of heavy investment in work could be linked to the perception of imbalance between work and nonwork domains. Although workaholics initially seem to represent an efficient type of hard workers in several work environments, they will probably demonstrate later the consequences of their lack of balance in life (Ng et al., 2007). This ambiguity potentially yields a danger, especially in organizational contexts: if hardworking styles were indiscriminately and systematically stimulated as good behavioral models to be replicated and rewarded, without any consideration of the different underlying nature of attitudes that can activate such behavior, there could be a real risk for some employees to get into an escalation towards a workaholic way of working; workaholism, in turn, can produce negative outcomes, like a problematic work–life interface, as observed in our study. Consequently, supervisors and managers should be aware of the real nature of workaholism and be able to recognize its behavioral signals among their employees.

Finally, since our results have shown a possible differentiation in the way heavy work investors perceive an interference among their life spheres, this knowledge might be efficiently used in the field of work–life balance policies, taking into account the distinctive characteristics of the different working styles. Organizations should offer a variety of programs, provided that not all the workers perceive conflict between work and life field in the same perspective or in the same direction. These initiatives could be used to present and promote a healthy approach to one’s job and, in turn, motivate a positive, balanced interface between work and nonwork spheres, thus creating an organizational environment where employees are more likely to develop an engaged working style.
